# Estrogen and progesterone attenuate CD4-positive immune cell traffic to the penumbra region of rat’s ischemic stroke brain

**DOI:** 10.22038/ijbms.2025.84415.18275

**Published:** 2025

**Authors:** Abutaleb Tamtaji, Sayyed Alireza Talaei, Homayoun Naderian, Mohammad Ali Atlasi, Nasim Alipour, Abolfazl Azami Tameh

**Affiliations:** 1 Anatomical Sciences Research Center, Institute for Basic Sciences, Kashan University of Medical Sciences, Kashan, Iran; 2 Physiology Research Center, Institute for Basic Sciences, Kashan University of Medical Sciences, Kashan, Iran; 3 Department of Medical Basic Sciences, MMS.C., Islamic Azad University, Mashhad, Iran

**Keywords:** CD4^ +
^, Estrogen, IL-1β, Progesterone, Stroke, tMCAO

## Abstract

**Objective(s):**

Stroke is an acute cerebrovascular disease with a high incidence, high disability rate, and high mortality. Stroke damages the integrity of the blood-brain barrier. Immune cells are concerned with multiple facets of ischemic stroke; peripheral immune cells, including neutrophils, T cells, B cells, and macrophages, infiltrate the ischemic brain tissue and are essential in regulating the progression of ischemic brain damage. The current study investigated the effects of estrogen and progesterone (PROG) hormones (E/P) on the expression of CD4^+^ and Gene expression of IL-1β (interleukin-1β) in MCAO rat models.

**Materials and Methods:**

Stroke was induced in male adult rats by transient middle cerebral artery occlusion (tMCAO). Rats were collected 24 hr after reperfusion, and then the doses of estrogen and progesterone were administered two hours after tMCAO. The expression of CD4^+^ using the immunohistochemistry (IHC) method and gene expression of IL-1β using the Real-time PCR in the ischemic penumbra of the male rat’s brain cortex were determined, and infarct volume was determined 24 hr after ischemia using TTC staining.

**Results:**

CD4^+^ and Gene expression of IL-1β were significantly increased in the Ischemia group compared to the control group. Also, E/P administration reduced infarct volume and CD4^+^ and gene expression of IL-1β compared to the Ischemia group.

**Conclusion:**

The results of the present study showed that induction of tMCAO altered the expression of CD4^+^ and gene expression of IL-1β in the ischemic penumbra. Moreover, E/P treatment could reverse these effects of stroke.

## Introduction

Ischemic stroke is a significant cause of human disability and mortality worldwide (1, 2). Following a cerebral artery occlusion, a region of the central nervous tissue infarcts in a matter of minutes, but the penumbra, or tissue around the center of the ischemia, is still at risk (3). One of the hallmarks of ischemic stroke is the disruption of the blood-brain barrier (BBB), which increases its permeability. Microglia, macrophages, neutrophils, and T lymphocytes interact directly or indirectly with BBB components and influence the integrity of the BBB following ischemic stroke. In addition, damage to the BBB allows the extravasation of neutrophils to the brain parenchyma, increasing the release of deleterious inflammatory mediators (4). Interleukin (IL)-1 is an inflammatory cytokine that plays a significant role in controlling immunological responses after an ischemic stroke and may be a target for stroke therapy. In experimental animal models of ischemic stroke, IL-1β rapidly rises in blood and brain, amplifying damage. In practical animal models, the IL-1 receptor antagonist (IL-1Ra) reduces the volume of ischemic infarcts (5). 

T lymphocytes are found adjacent to the infarct area within days after a stroke in post-mortem human samples. CD8^+^ T cells, CD4^+^ T cells, and NK (Natural Killer) T cells are recruited within 24 hr after the ischemic stroke, and the collection of these cells in the early inflammatory stage peaks 3 to 4 days after the injury. However, it is still indistinct if T cells present at the chronic stage following stroke are beneficial or detrimental to functional recovery (6).

Finding efficient neuroprotection that can save neurons in the penumbra is the main challenge in treating ischemic stroke. The ischemic damage extends to neighboring cells and leads to the creation of the transition area (ischemic penumbra) between the zone of the ischemic core and normal tissue. Therefore, during the first 24 hr following an ischemic stroke, research findings on the molecular mechanisms behind penumbra development and the expression of several signaling proteins in the penumbra are reviewed (7). 

Following middle cerebral artery occlusion (MCAO), treatment with exogenous progesterone and estrogen has been proven to protect the brains of male and female rodents from ischemic injury. Progesterone and estrogen have neuroprotective properties (8, 9) via their potent anti-inflammatory activities (10, 11). They are produced by the brain when steroidogenic enzymes are present (12, 13).

Despite several studies to elucidate the effects of estrogen and progesterone hormones on brain damage caused by cerebral ischemia, it is not known whether endogenously produced progesterone and estrogen enhance the brain’s resistance to ischemic insult. Based on this, the present study investigated the effects of 25 μg estrogen (E) and 10 mg progesterone (P) per kg body weight on the inflammatory factors IL-1β and CD4^+ ^and the infarct volume in a rat model of ischemic stroke.

## Materials and Methods

All animal procedures were conducted following the guidelines of the National Institutes of Health (NIH) approved by the Committee for the Care and Use of Laboratory Animals at Kashan University of Medical Sciences (KAUMS), Kashan, Iran (IR.KAUMS.medical.REC. 92158).

***Animals***

Male Wistar rats, weighing 250–300 g and 16 weeks old, were purchased from the KAUMS animal house. Animals were housed at 23±2 °C (55±5% humidity) with a light/dark period of 12 hr and had free access to standard laboratory water and feed. The animals were randomly divided into three groups: Control (Cnt), Ischemia (Isc), and EP-treated ischemia (EP).

***Drug preparation***

Twenty-five μg E and 10 mg P per kg body weight (Sigma, Germany) were diluted in pure ethanol and mixed in 500 μl sesame oil for hormone treatment. Rats in the EP group received the drug percutaneously immediately after MCAO induction. The dose of steroids and type of drug application have been previously reported to gain rapid physiologically high steroid plasma levels (E ~ 200 pg/ml, P ~ 70 ng/ml) and a maximum of short-term protection (14).

*Transient focal ischemia animal model and hormone treatment*

Transient focal cerebral ischemia was induced using occlusion of the middle cerebral artery (MCA) by the intraluminal filament method. In summary, rats were first anesthetized with 5% isoflurane and then kept under anesthesia with 3% isoflurane using an isoflurane evaporator (Eickemeyer, Germany). Cerebral blood flow (CBF) using Laser-Doppler flowmetry (Moor Instruments VMS-LDF2, UK) was monitored during surgery. The skull was thinned at a point located 4 mm lateral and 2 mm posterior to the Bregma on both the left and right hemispheres. Fiber optics were placed at the mentioned points to measure the CBF of the MCA as perfusion units. The neck’s common carotid artery (CCA) was exposed, and its end was closed. The monofilament (Doccol, USA) was introduced through the CCA into the lumen of the internal carotid artery (ICA) until a significant reduction in CBF of MCA was observed. Only rats with more than a 50% reduction in blood flow compared to baseline CBF levels before the induction of MCAO were entered in this study. After 60 min of ischemia induction, the catheter was cautiously drawn out to restore reperfusion, and rats were permitted to recover from anesthesia fully. CBF was constantly observed during ischemia (60 min), and monitoring persisted for 10 min after withdrawing the catheter. The Control rats experienced a similar surgery, but the catheter was not inserted. For hormone treatment, rats in the EP group received the drug percutaneously immediately after MCAO induction. The Control group received a single dose of ethanol/sesame oil mixture (14-16).

***2,3,5-triphenyl-tetrazolium-chloride (TTC) staining***

We employed 2,3,5-Triphenyltetrazolium chloride (TTC) staining to define the infarction volume. Twenty-four hours after ischemia, the rats were deeply anesthetized with 10% chloral hydrate (500 mg/kg body weight, intraperitoneal). After euthanizing the animals, the brains were removed from the skulls and sliced into 2 mm-thick coronal sections using a brain matrix (Zivic Instruments, USA). The sections were immersed in a 2% TTC solution in PBS (phosphate-buffered saline) for ten minutes at 37 °C. Then, brain sections were fixed with 4% paraformaldehyde in PBS. The stained tissue sections were examined after 12 hr, and the infarct size was determined by image analysis and described as the percentage of the entire cerebral cortex tissue. Infarcted areas of all sections were measured (mm2), and infarct volumes were calculated by multiplying the sum of the infarct areas by the distance between sections (2 mm) (8, 17).

***Real-time PCR***

Samples were taken from the ischemic penumbra of the cortex to analyze the expression of IL1-β as target genes and hypoxanthine phosphoribosyl transferase (HPRT) as a reference gene. According to the manufacturer’s instructions, total RNA was separated from rat brain tissue in the penumbra area of the cerebral cortex using RNX Plus Solution (Sinaclon, Iran). A spectrophotometer (NanoDrop, USA) was used to determine the purity and concentration. Complementary DNA (cDNA) was synthesized using cDNA synthesis kits (Takara Bio) following the manufacturer’s guidelines, and the product was stored at -20 °C until use. SYBR green PCR master mix was used to amplify the desired fragments by real-time PCR method using specific primer sets for HPRT as the housekeeper gene: Forward GCTCGAGATGTCATCAAGGAGA/ Reverse TCAGCGCTTTAATGTAATCCAGC and IL-1β as the target gene: Forward CTGTGACTCGTGGGATGATG/ Reverse GGGATTTTGTCGTTGCTTGT. Results were normalized to the HPRT mRNA level, and fold-change was analyzed using the 2^-ΔΔCt ^method. The specificity of primers was checked using the NCBI BLAST tool. 

*Tissue preparation*

Animals were anesthetized with chloral hydrate and perfused, culminating in the heart being fixed with 4% paraformaldehyde. Brains were removed from the skull and post-fixed in the same fixative for 48 hr. After fixation, brain tissues were dehydrated by ascending ethanol concentrations from 70% to 100%. Then, the tissues were immersed in paraffin, and using microtomes, coronal tissue sections with a thickness of five micrometers were prepared from paraffin blocks and mounted on Poly-L-lysine-coated slides (*18*). Animals were deeply anesthetized with chloral hydrate and perfused transcardially with 4% paraformaldehyde. Brains were removed from the skull and post-fixed in the same fixative for 48 hr. After fixation, brain tissues were dehydrated by ascending ethanol concentrations from 70% to 100%. Then, the tissues were immersed in paraffin. Coronal brain sections with a thickness of five micrometers were prepared from paraffin blocks using a microtome and mounted on Poly-L-lysine-coated slides (18, 19). 

*Immunohistochemistry*

IHC kit (UltraTek HRP-Anti Polyvalent, ScyTek Biotech Life Sciences) was used for immunohistochemistry. Brain sections were deparaffinized using xylene, rehydrated, and washed with 0.1 M saline phosphate buffer (PBS). The antigen retrieval process was performed on slides with preheated citrate buffer at 95 °C for 15 min, then washed with PBS three times after cooling at room temperature. In the next step, to inhibit endogenous peroxidase, the sections were treated with 30% hydrogen peroxide in methanol for 10 min and washed again with PBS three times. Sections were incubated with the primary antibody Anti-CD4 (Abcam, Germany) diluted in serum overnight at 4 °C, then washed with PBS. Linker incubation (Diagnostic BioSystems-PVP1000D) of sections was performed for 15 min, then washed with PBS. Then, sections were incubated with 100 µl of DAB (3, 3′-Diaminobenzidine) solution (ScyTek-ACV999) and were washed with water. Finally, sections were counterstained with Harris hematoxylin for 10 sec, washed with water, and dehydrated with ascending ethanol concentrations. In the end, to clarify the tissues, sections were placed in xylene twice for five minutes each time and mounted with coverslips (17, 18, 20). 

*Quantitative and statistical analysis*

Slides were examined using a light microscope (Olympus BX51, Japan) connected to a camera (Olympus DP12, Japan) with an ND25 and an OP filter. Images of samples were taken at ×100. The numerical surface density of neurons on the tissue sections with a magnification of ×100 was estimated using the counting frame and instrument in stereology. Data were analyzed using GraphPad Prism 9 software, and one-way ANOVA was used for analysis of variance to determine overall significance between Controls (n=10), Ischemia (n=8), and EP-treated ischemia (n=7) groups. Data are given as mean ± SEM and P-values less than 0.05 were considered statistically significant. It should be mentioned that three samples from the cortex of each animal were taken for the gene expression study.

## Results

**Figure 1:**
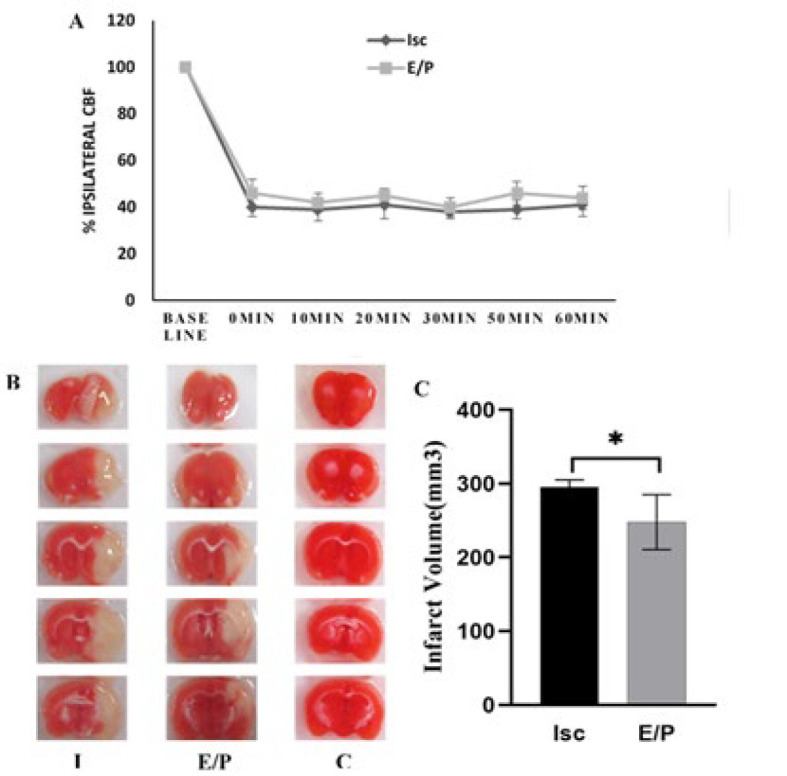
cBF course and infarct volume in experimental animals

Results from Laser-Doppler monitoring showed that after induction of tMCAO, only rats with a CBF reduction of more than 50% compared to baseline values were included in the study. As shown in Figure 1A, mean values of the Isc and E/P groups decreased by ~60% compared to baseline values, and this value remained unchanged during the ischemia period. Ischemia significantly reduced the maximum scoring from 18 points (Cnt) to 10.6 points (Isc), and hormone treatment significantly enhanced the neurological scores compared to the Isc group (*P*<0.05) (*P*<0.05) (Figure 1C).

***Infarct volume***

Using TTC staining, the infarct volume of brain sections was defined 24 hr after induction of ischemia. Red-colored areas in brain sections stained with TTC correspond to living tissues, and white-colored areas indicate infarcted tissues. There was no detectable infarct area in the Cnt group, while an extensive infarct area was detected in the animals of the Isc group (Figure 1B). EP treatment significantly reduced the infarct area’s volume from 248.8 to 408.1 mm^3^ compared to the Isc group (P<0.05), as shown in Figure 1C.

***CD4*** ^+^ ***expression in the ischemic penumbra and the effect of E/P treatment***

**Figure 2:**
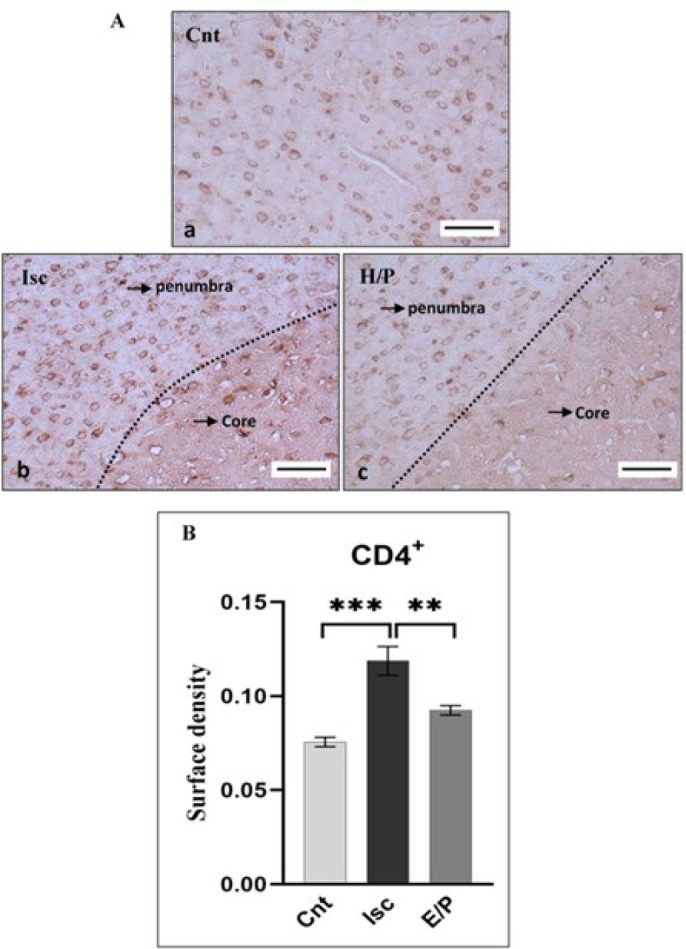
Evaluation of CD4+ expression in the cortical region of the brain following ischemia induction in rats and the impact of estrogen/progesteron (E/P) treatment

In the Ischemia group, the expression of CD4^+^ increased compared to the control group (*P*<0.01 and *P*<0.0001, respectively). Moreover, the expression of CD4^+^ in the EP-treated ischemia group decreased compared to the Ischemia group (*P*<0.05). The expression of CD4^+^ in the ischemic penumbra in the EP-treated ischemia group decreased. Also, no significant difference was observed between the control and EP-treated ischemia groups (Figure 2A and 2B).

***IL-1β expression in the ischemic penumbra and the effect of E/P treatment***

**Figure 3:**
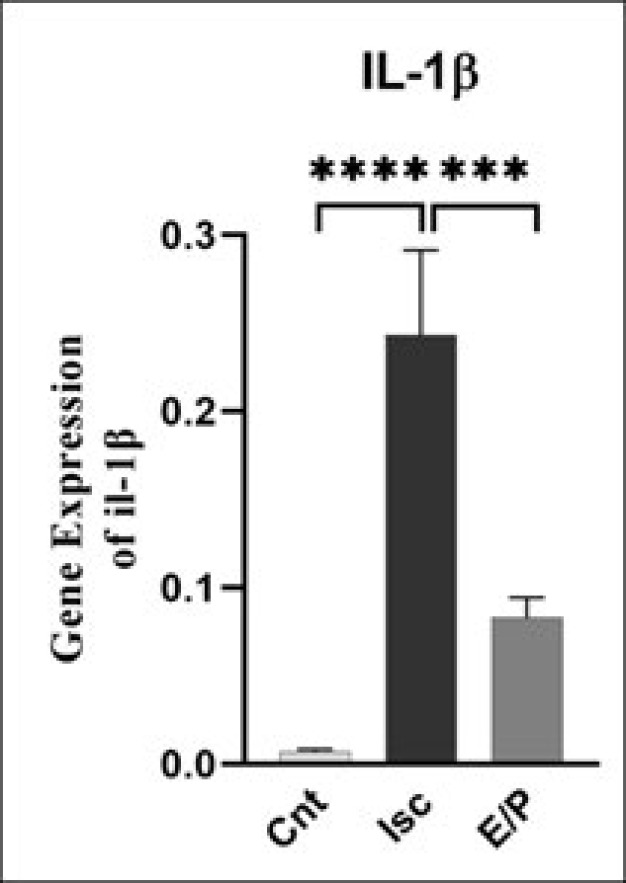
mRNA expression level of IL-1β gene in experimental groups

In the ischemia group, the mRNA expression of the IL-1β gene increased compared to the control group (*P*<0.01). Moreover, the IL-1β gene expression in the EP-treated ischemia group decreased compared to the Ischemia group (*P*<0.05). The IL-1β gene expression in the ischemic penumbra in the EP-treated ischemia group decreased. Also, no significant difference was observed between the control and EP-treated ischemia groups (Figure 3).

## Discussion

In the present study, we exhibited the importance of exogenous estrogen and progesterone in cerebroprotection of male rats during the early critical phase after transient focal cerebral ischemia induced by MCAO. Following MCAO, the expression of IL-1β and CD4+ increased in the penumbra region of the cerebral cortex, and treatment with estrogen and progesterone modulated the adverse effects of ischemia on the cerebral cortex and decreased infarct size. 

When a CNS injury occurs, the detrimental consequences of IL-1β are visible, and the cytokine levels are elevated. TNF, interleukin (IL)-1, and IL-23, among the inflammatory cytokines generated by macrophages, have been demonstrated to have a substantial role in brain injury and neural dysfunctions (21). Raymond Wong, in 2019, stated that Interleukin-1 (IL-1) is a crucial mediator of the neurodegeneration induced by cerebral ischemia (stroke) in rodents (22), and the processes underlying IL-1’s effects on neuroinflammation in response to stroke are complex (23-25). However, it is stated that IL-1 activates the brain endothelium, resulting in the destruction of endothelial tight junctions, which compromises the integrity of the blood-brain barrier and may affect neutrophil infiltration of the brain tissue and subsequent neuronal damage, which was confirmed by our results with the increase of CD4^+^ immune cells in the penumbra area. Additionally, IL-1 is a strong inducer of neuronal chemokines, which may impact the severe neuro-inflammatory and microglia responses. Thus, by affecting every brain cell, IL-1 can mediate the central inflammatory response (23). When the brain is injured by cerebral ischemia, neutrophils are among the first cells to infuse the brain, boosting inflammatory responses that further disrupt the BBB, cause cerebral edema and brain damage, and increase the infarct volume finally (26). Neutrophils, platelets, and endothelin-1 expression are all stimulated by IL-1, and this could change cerebral perfusion (27). In the penumbra or peri-infarct region, microglial cells are activated. Microglia multiply when triggered, in addition to changing their activation status due to cerebral damage (28). The innate immune system is the body’s initial defense against damage and pathogen invasion. The innate immune response, which involves the invasion and activation of peripheral and brain innate immune cells, is involved in the early stages of post-ischemic stroke (29). In conclusion, in an experimental ischemia stroke model, ischemic stroke may cause long-term T cell infiltration and activation in the brain. 

Up to one month after an ischemic stroke, there was a considerable increase in CD4^+^ and CD8^+^ T cells in the peri-infarct area. The extended activation of brain-invading T lymphocytes may influence the neurons after cerebral ischemia (29). T and B lymphocytes have a variety of functions in the pathophysiology of stroke (21). Several data suggest that T lymphocytes encourage brain damage during the initial stages of a stroke. Brait VH, in 2012, states that CD4^+^ T cells and NK cells invade the brain 24 hr after an ischemic stroke. Therefore, T cells may be a useful therapeutic target for stroke (21). Another study reported the function of activated/effector CD4^+^ T cells in the nervous system; specifically, a sudden, uncontrolled influx of activated CD4^+^ T cells into the nervous system can exacerbate acute CNS damage after traumatic injury (30), and this could be a result of magnificent leucocyte loss days after stroke onset. The pro-inflammatory cytokines secreted by CD4^+ ^cells have a strong cytotoxic effect on the endothelium. The vascular endothelium may become damaged due to this T-cell subset expansion changing from an antithrombotic and anticoagulant state to a prothrombotic and procoagulant state (31). Since inflammation plays a significant role in the pathophysiology of stroke, it is a desirable therapeutic target (32). 

Following cerebral ischemia, gender differences in treatment outcomes have frequently been noted. Although there has been little evidence of this in human outcome studies, progesterone and estrogen (9) are thought to offer protection in the immediate post-injury period, suggesting that females have an advantage. This steroid hormone (PROG) may have pharmacotherapeutic benefits in several experimental injury models, including stroke and trauma (33). Additionally, it has repeatedly been demonstrated that PROG and estrogen decrease the inflammatory response. Also, these two hormones dramatically reduced the loss of neural tissue. They aided functional recovery from traumatic brain damage in the initial stages of neurological injury, such as traumatic brain injury (TBI). According to some evidence, PROG and estrogen protect against brain damage and cerebral infarction by preventing inflammatory responses and BBB disruption. However, the precise mechanism is still unknown and has to be studied further (33). Results obtained from studies conducted in this field indicate that estrogen and progesterone treatment can be related to the dose and duration used, as well as to differential effects related to gender or age. Hormone exposure at high doses can intensify infarction volume after MCAO (34). It is also stated that progesterone is not neuroprotective in the ischemic female rat brain when administered as a single dose (35). Therefore, considering this issue, we used a combined dose of estrogen and progesterone in the present study. On the other hand, it stated that the shortage of neuroprotection and increased brain injury also conflict with the data from the study that reports the beneficial effects of progesterone therapy in very young animals (36).

In both permanent and temporary rodent models of MCAO, progesterone and estrogen have decreased infarct volume, edema, global inflammation, and behavioral impairments compared to the control group. Progesterone has an anti-inflammatory impact, but when estrogens and progesterone are together, this effect is synergic. These hormones concerning the cerebral vascular response to IL-1b have a strong anti-inflammatory effect, whereas progesterone reduces cerebral edema by accelerating the production of pro-inflammatory cytokines (IL-1β, TNF-a) (37). Numerous immune cells, including T cells and NK cells, have progesterone receptors (11). Progesterone has both immunomodulatory and anti-inflammatory actions by influencing immune cells, in addition to its neuroprotective properties. Specific receptors in various immune cells, including lymphocytes, monocytes, macrophages, and DCs, mediate these actions (38). Also, estrogen receptors (ERα) are expressed in different immune cells, including CD4^+^ and CD8^+^ T cells, NK cells, and macrophages (38). These studies and the results of the present study bring us to the conclusion that the significant reduction of CD4^+ ^cells in the ischemic penumbrae after steroid treatment could define a regulatory role for estrogen and progesterone in immune cell migration to this region.

A) Laser-Doppler measurements were conducted to assess cerebral blood flow (cBF) in the territory of the middle cerebral artery (MCA) on the ipsilateral side, both before and during the first hour after transient middle cerebral artery occlusion (tMCAO). This method allows for real-time monitoring of blood flow dynamics, providing insights into the immediate effects of MCA occlusion. B) Tissue slices stained with TTC (triphenyltetrazolium chloride) were analyzed. In these slices, the areas of undamaged tissue appear red, indicating viable brain regions, while the infarcted tissue is shown in a lighter color, reflecting necrotic areas resulting from the ischemic event. This staining technique is commonly used to visualize areas of infarction and assess the extent of brain injury. C) A quantitative analysis of cerebral infarct volumes was performed, revealing significant differences between experimental groups. The statistical significance of differences between the groups is indicated as * *P*<0.01.

(A) Immunohistochemical (IHC) analysis was conducted to evaluate the expression of CD4^+^ cells following transient middle cerebral artery occlusion (tMCAO) across different experimental groups: Cnt (control), Isc (ischemia), and steroid hormone (EP-treated ischemia). This analysis focused on the ischemic penumbra area of the cerebral cortex. The immunoreactivity was visualized using diaminobenzidine (DAB), and scale bars in the images represent 100 μm. (B) Quantitative assessment of CD4^+^ cell number per unit area showed a significant increase after ischemia, which is reduced after EP hormone treatment. The statistical significance of differences between the groups is indicated as ** *P*<0.01 and *** *P*<0.001.

The expression of the IL-1β gene was significantly elevated in the brain cortical region of ischemic animals compared to the control group. In contrast, treatment with EP reduced IL-1β expression by more than half in the ischemic animals. This suggests that the effect of estrogen/progesteron (E/P) treatment on reducing IL-1β expression is statistically significant, highlighting its potential therapeutic efficacy in managing inflammation following ischemic events. The groups were classified as Cnt for control, Isc for ischemia, and EP for ischemia treated with EP. The statistical significance of differences between the groups is indicated as follows: *** *P*<0.001 and **** *P*<0.0001.

## Conclusion

Our study demonstrated that estrogen and progesterone significantly reduce the expression of interleukin-1β and CD4+ in the cortical region of the rats’ brain following ischemia. During ischemic stroke, these hormones play a protective role in the brain by decreasing inflammatory factors, primarily through their modulatory effects on infiltrating immune cells in the injured area. However, further studies should be conducted to clarify the molecular mechanisms involved in the interactions between steroids and infiltrating immune cells in the penumbra region.

## Data Availability

The datasets generated during and/or analyzed during the current study are available from the corresponding author upon reasonable request.

## Declaration

We have not used any AI tools or technologies to prepare this manuscript.
